# Is postoperative non-weight-bearing necessary? INWN Study protocol for a pragmatic randomised multicentre trial of operatively treated ankle fracture

**DOI:** 10.1186/s13063-021-05319-0

**Published:** 2021-05-27

**Authors:** Ramy Khojaly, Ruairí Mac Niocaill, Muhammad Shahab, Matthew Nagle, Colm Taylor, Fiachra E. Rowan, May Cleary

**Affiliations:** 1 Department of Trauma and Orthopaedic Surgery, University Hospital Waterford, Waterford, X91 ER8E Ireland; 2 Department of Surgery, Royal College of Surgeons in Ireland, Dublin, D02 YN77 Ireland; 3 Department of Orthopaedic Surgery, University College Cork, Cork, T12 YN60 Ireland; 4 Department of Trauma and Orthopaedic Surgery, Cork University Hospital, Cork, T12 DFK4 Ireland

**Keywords:** Ankle fracture, Osteosynthesis, Fracture fixation, Open reduction and internal fixation, Weight-bearing, Immobilisation, Cast, Orthosis, Walking boot, Randomised controlled trial

## Abstract

**Background:**

Postoperative management regimes vary following open reduction and internal fixation (ORIF) of unstable ankle fractures. There is an evolving understanding that extended periods of immobilisation and weight-bearing limitation may lead to poorer clinical outcomes. Traditional non-weight-bearing cast immobilisation may prevent loss of fixation, and this practice continues in many centres. The purpose of this trial is to investigate the safety and efficacy of immediate weight-bearing (IWB) and range of motion (ROM) exercise regimes following ORIF of unstable ankle fractures with a particular focus on functional outcomes and complication rates.

**Methods:**

A pragmatic randomised controlled multicentre trial, comparing IWB in a walking boot and ROM within 24 h versus non-weight-bearing (NWB) and immobilisation in a cast for 6 weeks, following ORIF of all types of unstable adult ankle fractures (lateral malleolar, bimalleolar, trimalleolar with or without syndesmotic injury) is proposed. All patients presenting to three trauma units will be included. The exclusion criteria will be skeletal immaturity and tibial plafond fractures. The three institutional review boards have granted ethical approval. The primary outcome measure will be the functional Olerud-Molander Ankle Score (OMAS). Secondary outcomes include wound infection (deep and superficial), displacement of osteosynthesis, the full arc of ankle motion (plantar flexion and dorsal flection), RAND-36 Item Short Form Survey (SF-36) scoring, time to return to work and postoperative hospital length of stay. The trial will be reported in accordance with the CONSORT statement for reporting a pragmatic trial, and this protocol will follow the SPIRIT guidance.

**Discussion:**

Traditional management of operatively treated ankle fractures includes an extended period of non-weight-bearing. There is emerging evidence that earlier weight-bearing may have equivocal outcomes and favourable patient satisfaction but higher wound-related complications. These studies often preclude more complicated fracture patterns or patient-related factors. To our knowledge, immediate weight-bearing (IWB) following ORIF of all types of unstable ankle fractures has not been investigated in a controlled prospective manner in recent decades. This pragmatic randomised-controlled multicentre trial will investigate immediate weight-bearing following ORIF of all ankle fracture patterns in the usual care condition. It is hoped that these results will contribute to the modern management of ankle fractures.

**Trial registration:**

ISRCTN Registry ISRCTN76410775. Retrospectively registered on 30 June 2019.

**Supplementary Information:**

The online version contains supplementary material available at 10.1186/s13063-021-05319-0.

## Administrative information

The order of the items has been modified to group similar items (see http://www.equator-network.org/reporting-guidelines/spirit-2727-statement-defining-standard-protocol-items-for-clinical-trials/).

**Table:** 

Title {1}	Is Postoperative Non-Weight-bearing Necessary? (INWN), study protocol for a pragmatic randomised multicentre trial of operatively treated ankle fracture.
Trial registration {2a and 2b}	‘retrospectively registered’ ISRCTN - ISRCTN76410775 30/06/2019
Protocol version {3}	Version (3) June 2019
Funding {4}	Investigator-initiated and funded
Author details {5a}	Ramy Khojaly^1,2^, Ruairi Mac Niocaill^1^, Muhammad Shahab^1^, Matthew Nagle^3^, Colm Taylor^3^, Fiachra E. Rowan^1^, May Cleary^1,5^. 1. Department of Trauma and Orthopaedic Surgery, University Hospital Waterford, Waterford, X91 ER8E, Ireland 2. Department of Surgery, Royal College of Surgeons in Ireland, Dublin, D02 YN77, Ireland. 3. Department of Trauma and Orthopaedic Surgery, Cork University Hospital, Cork, T12 DFK4, Ireland. 4. Department of Orthopaedic Surgery, University College Cork, Cork, T12 YN60, Ireland
Name and contact information for the trial sponsor {5b}	Ramy Khojaly, MBBS, MRCSI, MSc Tr & Orth. Orthopaedic Registrar and Clinical Lecturer in Orthopaedic Surgery, Department of Trauma and Orthopaedic Surgery, University Hospital Waterford.Department of Surgery, Royal College of Surgeons in Ireland Honorary Orthopaedic clinical lecturer at the University College Cork. Email: ramykhojaly@rcsi.com Telephone: +353-51 842 198
Role of sponsor {5c}	There is no sponsor

## Introduction

### Background and rationale {6a}

Ankle fractures are common and affect young adults as well as the elderly [1]. An unstable ankle fracture treatment typically involves surgical fixation, immobilisation and modified weight-bearing for 6 weeks. Immobilisation can have implications for patient function and may reduce independence, mobility and return to work.

There is emerging evidence that extended periods of immobilisation and weight-bearing limitation may lead to poorer outcomes [2]. Traditional non-weight-bearing (NWB) cast immobilisation periods of six or more weeks were used to protect the soft tissue envelope and osteosynthesis [3]. Newer trends in earlier mobilisation compete with traditional NWB doctrine, and weak consensus exists as to the best postoperative strategy [4, 5]. This could be explained by the contradicting literature regarding the assessment of weight-bearing regimens following ankle fracture fixation [2, 6–12].

Some studies have investigated early mobilisation without weight-bearing, early weight-bearing (EWB) or immediate weight-bearing (IWB) following fixation of ankle fractures, some of which reported favourable outcomes [2, 6–10], and others raised concerns of increasing complication rates [9, 11, 13].

Between 1986 and 1993, Ahl et al. performed four small RCTs, with a sample size ranging from only 40 to 53 patients in each trial. They compared IMW in a cast from day 1, in the first two trials, or EWB in a cast after 1 week, in the third and fourth trials, to NWB for 7 weeks after surgical fixation of lateral malleolar and bimalleolar fracture. The results showed higher surgical site complications in the IWB group than in the EWB group, and the authors recommended delaying weight-bearing until wound healing was complete.

The largest published trial in the subject by Dehgan et al. included 110 patients. They investigated early weight-bearing from 2 weeks versus late weight-bearing. Although the trial has failed to prove earlier return to work (the primary outcome) for EWB group patients, the authors recommended early weight-bearing based on early improved functional outcome and lack of increased complications rate [2]. Most recently, Smeeing et al. attempted a multicenter RCT to investigate three groups of patients: unprotected non-weight-bearing, protected weight-bearing as tolerated and unprotected weight-bearing following surgical fixation of ankle supination external rotation injury only. This trial was discontinued before completing the target number due to lack of funding and the slow recruitment process. The published post hoc analysis included a total of 115 recruited patients in all three groups. The authors concluded that unprotected weight-bearing and mobilisation improved short-term functional outcomes without increasing complication rate [10].

Our null hypothesis was that immediate weight-bearing and early mobilisation protocols are not superior to or the same as non-weight-bearing and immobilisation protocols.

### Objectives {7}

The primary objective is to determine whether immediate protected weight-bearing and ankle range of motion post-open reduction and internal fixation of unstable ankle fractures improve functional outcomes compared to postoperative ankle immobilisation in a non-weight-bearing cast; second, to determine whether the rate of complications, such as wound infection and fixation failure, with immediate weight-bearing and ROM, is comparable to the rates with the usual postoperative protocols; and finally, to determine the cost-effectiveness of this treatment method, which can be determined by the analysis of patients’ ability to return earlier to work and the detailed cost of either intervention, including the length of hospitalisation.

### Trial design {8}

The study will be a prospective, pragmatic randomised controlled trial (p-RCT), unblinded with participants allocated in a 1:1 ratio to one of two parallel groups. Patients will be randomised using computerised block randomisation (twenty patients per block). The study is multicentre and will include three major orthopaedic centres in Ireland.

## Methods: participants, interventions and outcomes

### Study setting {9}

This pRCT will be conducted at three academic trauma units at three different urban centres in Ireland. Each centre serves a referral population of >500,000 and receives all grades of trauma from urban and rural environments on a 24-h basis. A trauma team is on call daily and includes two trainee surgeons and a consultant orthopaedic surgeon. Surgeries are performed in part or in total or supervised by consultant orthopaedic surgeons. Regional and general anaesthesia is used at the discretion of the anaesthetist. Ankle fracture surgery is conducted on both a day case or an overnight basis. Ward-based physiotherapy is provided daily to facilitate early discharges. All hospital personnel contributing to the recruitment and patient pathways in this trial will undergo training in the study’s objectives and methodology.

### Eligibility criteria {10}

#### Inclusion criteria

The inclusion criteria include all skeletally mature (closed distal tibial physis), acute ankle fractures treated with anatomical reduction and stable internal fixation, including AO/OTA 44A1.3 to 44A3.3, 44B and 44C.

- Isolated lateral malleolar fractures
- Isolated medial malleolar fractures
- Bimalleolar fractures
- Trimalleolar fractures
- Syndesmosis injuries that have been surgically fixed with either screw or tightrope
- Closed fractures and grade I or grade II open fractures

#### Exclusion criteria

The following are the exclusion criteria:

- Skeletal immaturity (open distal tibial physis)
- Gustilo grade III open fractures
- Tibial plafond fractures
- Polytraumatised patients
- Non-ambulatory status before injury
- Expected insufficient stable fracture fixation with standard surgical technique (e.g. comminuted fractures or poor bone quality and screws fixation)
- Pre-existent cognitive disability, neurological disease or inability to comply with non-weight-bearing mobilisation
- Grossly comminuted fragility fractures

### Who will take informed consent? {26a}

All patients admitted to the hospital with ankle fracture (AO/OTA 44A1.3 to 44A3.3, 44B and 44C) deemed appropriate for surgical intervention will be asked by the admitting trainee or consultant surgeon to participate in the trial, provided with a patient information leaflet (Additional file 1) and given time to read the document and ask questions. If the patient agrees to enter the trial, they will sign the consent form in the admitting doctor’s presence on the morning or the night before their surgery.

### Additional consent provisions for collection and use of participant data and biological specimens {26b}

Not applicable.

### Interventions

#### Explanation for the choice of comparators {6b}

Traditional non-weight-bearing (NWB) cast immobilisation is a common practice in many centres, and this protective protocol might not be necessary.

#### Intervention description {11a}

In accordance with a pragmatic study, the surgical approach and choice of the implant will be at the surgeon’s discretion. Surgeons may or may not be authors in the study. Surgical practice at the three institutions is to achieve anatomical reduction and rigid fixation. The commonly used osteosynthesis system for fixation is the small fragment system, with a one-third tubular plate commonly used. The use of locking mode is not routinely used. Other systems are also available. All patients will be assessed by a physiotherapist for gait stability and provided with walking aids according to the randomisation. Patients in the walking boot group (group A) will be instructed to weight-bear as tolerated immediately with or without walking aids for balance. Patients in the NWB group (group B) will be instructed to strictly bear non-weight using crutches or frames for a total of 6 weeks. Group A will be instructed to remove the walking boot four times a day at minimum to perform ankle range of motion exercises until they attend outpatient physiotherapy following their first postoperative visit. According to their grouping, all patients will receive a postoperative care information sheet (Additional files 2 and 3).

Patients will be allocated randomly to one of the following two groups:

Group A:

- Will receive a walking boot orthosis postoperatively in theatre and allowed weight-bearing as tolerated and range of motion (ROM) exercises immediately.
- Elevation of the affected foot in the first 2 weeks will be encouraged to reduce swelling.
- The first follow-up appointment will be after 2 weeks. This visit is for surgical site inspection, removal of sutures, check X-ray and referred to physiotherapy to continue ROM exercises and weight-bearing as tolerated progressing to full weight-bearing.

Group B:

- Will receive full below-knee cast postoperatively in theatre and prevented weight-bearing for 6 weeks.
- Elevation of the foot in the first 2 weeks will be encouraged to reduce swelling.
- The first follow-up appointment was after 2 weeks. This visit is for surgical site inspection, removing of sutures, checking of X-ray and re-application of the entire below-knee cast.
- After 6 weeks, the second follow-up is for removal of cast and referral to physiotherapy to commence ankle ROM exercises and weight-bearing as tolerated progressing to full weight-bearing.

### Criteria for discontinuing or modifying allocated interventions {11b}

The trial will be terminated early if a 20% complication rate (wound complications and fixation failure) is detected in either treatment group [10, 14].

### Strategies to improve adherence to interventions {11c}

#### Surgeon

Surgical treatment for all participants will remain unchanged in both groups, and all surgeons have agreed to participate in the trial.

#### Participant

In the postoperative setting on the ward and before discharge, a physiotherapist will reinforce the patient’s role in the trial and provide them with information leaflet appropriate to their grouping. At subsequent outpatient follow-up visits, patients will be reminded of the trial. The trial case report form (Additional file 4) will record if the patients have received outpatient physiotherapy.

### Relevant concomitant care permitted or prohibited during the trial {11d}

The choice of and duration of DVT prophylaxis will be at the surgeon’s discretion.

### Provisions for post-trial care {30}

None.

### Outcomes {12}

The primary outcome measure is the functional Olerud-Molander Ankle Score (OMAS) at 6 weeks postoperatively. This score ranges from 0 to 100, with 100 representing normal ankle function [15]. Secondary outcome measures include complication rate (infection and fixation failure), the entire arc of ankle motion (plantar flexion and dorsal flection) in degrees using a goniometer; RAND 36-Item Short Form Survey scoring (SF-36), from the RAND corporation [16]; the time needed to return to work in days; and postoperative hospitalisation length in days.

#### Follow-up

Patients will be followed up in an outpatient setting at 2 weeks, 6 weeks, 12 weeks, 6 months and 1 year postoperatively. At each visit, the OMAS and RAND-36 health questionnaire will be collected. Surgeons who may or may not be authors in the study reviewing patients in either group will also, at each follow-up appointment, complete a case report form that was created by the trial team (Additional file 4). This record includes surgical site assessment, any complication, X-ray evaluation, ankle range of motion (using goniometry), information regarding return to work, confirmation of physiotherapy referral and confirmation of the collection of OMAS and RAND-36 questionnaire.

### Participant timeline {13}

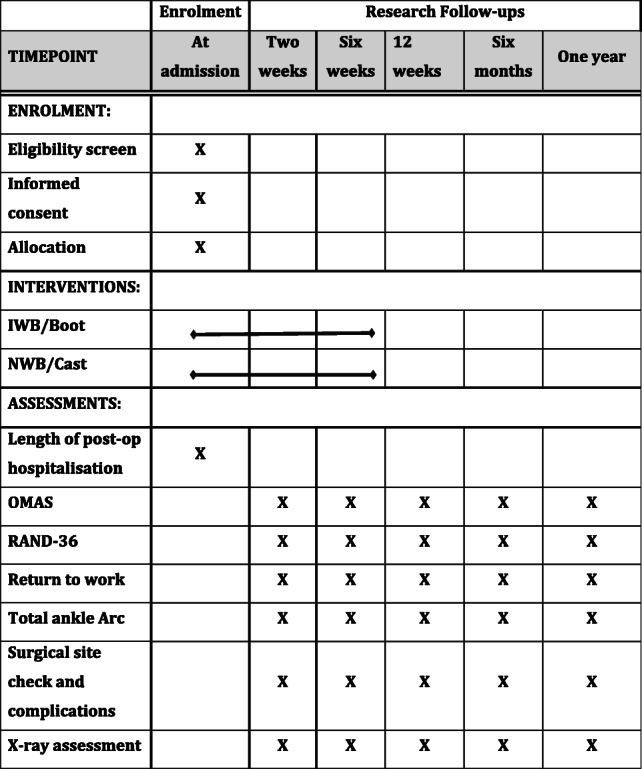



Standard Protocol Items: Recommendations for Interventional Trials (SPIRIT) figure of enrolment, interventions and assessments. *IWB* immediate weight-bearing, *NWB* non-weight-bearing, *OMAS* Olerud Molander Ankle Score, *RAND-36* 36-item Short-Form Health Survey

### Sample size {14}

An a priori power analysis for the superiority of treatment with immediate weight-bearing and ROM will be conducted for this hypothesis. To detect a clinically significant 10-point difference in the Olerud and Molander Ankle Score (OMAS) at 6 weeks, with a standard deviation of 19 [17–20], alpha = 0.05 and β=0.20 (80% power) and two-sided test and a maximum loss of follow-up of 20% (N=145), this was rounded to 160 to support the block randomisation (20 block size), and a sample size of 80 per group is necessary.

### Recruitment {15}

All patients admitted to the hospital with ankle fracture (AO/OTA 44A1.3 to 44A3.3, 44B and 44C) deemed appropriate for surgical intervention will be asked by the admitting trainee or consultant surgeon to participate in the trial, provided with a patient information leaflet (Additional file 1) and given time to read the document and ask questions.

### Assignment of interventions: allocation

#### Sequence generation {16a}

An online computer-generated block randomisation list (20 patients per block) will be created at the start of the trial via the website http://www.randomization.com. This list has a unique number that is stored safely and can be double-checked. Details of the randomisation block are provided in a separate document that is unavailable to those who enrol participants or assign interventions.

#### Concealment mechanism {16b}

Upon skin closure, a circulating theatre nurse, who is not part of the trial team, will consult the randomised block database, which is kept secure and password-protected. The nurse will inform the surgical team that a walking boot (group A) or a cast (group B) is to be applied. The patient’s details will be entered into the database, and they will be assigned a trial number. The surgeons will be blinded to the intervention until fracture fixation is complete.

#### Implementation of randomisation {16c}

An independent statistician will create the computer-generated block randomisation list. All patients admitted to the hospital with an ankle fracture for surgical intervention will be asked by the admitting trainee or consultant surgeon to participate in the trial. As described in details above (item 16b), a circulating theatre nurse will assign participants to intervention.

### Assignment of interventions: blinding

#### Who will be blinded {17a}

We understand there is some unavoidable risk of bias to this particular type of RCT where the intervention is impossible to blind as both cast and boot are visible. This has been a known weakness in all previous similar RCTs. Furthermore, in line with the pragmatic trial design, the patient’s care must follow the routine hospital follow-up procedure. It was not feasible to introduce independent assessors to mitigate such bias. On the other hand, patient-reported outcome measures (OMAS and RAND-36) are completed solely by patients, and the assessor has no role in these data. Additionally, to reduce the risk of bias, the surgeon will be blinded until the surgical procedure is complete.

#### Procedure for unblinding if needed {17b}

This RCT is unblinded.

### Data collection and management

#### Plans for assessment and collection of outcomes {18a}

The Olerud-Molander Ankle Score (OMAS) is a commonly used PROM in ankle fracture research, and often, it is the primary outcome measure [2, 6, 7, 12, 21]. This questionnaire was developed in 1984 to evaluate patients’ function with ankle fractures [15]. A recent systematic review assessing patient-reported outcome measures used for adults with an ankle fracture found that OMAS has sufficient levels of reliability, internal consistency and construct validity [22]. The Short Form (SF-36) Survey is the most widely used health-related quality of life measure in research to date because of its history, reliability and validity [23, 24].

At each follow-up visit, the OMAS and the RAND-36 will be collected from the participants by outpatient clinic nurses. The attending orthopaedic consultant or NCHD fills up a case report form, which includes documentation of the following information: surgical site assessment and complication, X-ray evaluation, ankle full arc measure (goniometry), information regarding return to work, confirmation of physiotherapy referral and confirmation of collection of OMAS and RAND-36 Health survey.

#### Plans to promote participant retention and complete follow-up {18b}

We have developed a patient tracking system to allow researchers to monitor follow-up carefully. As part of this system, a weekly list of expected patients is provided to the research nurse in the OPD, and this list is reviewed daily to record attendance. Suppose a patient is absent from the clinic. In that case, another appointment for the following week will be arranged, the RAND-36 and the OMAS score will be posted to the patients with prepaid envelope enclosed and the patient will be contacted to encourage follow-up.

#### Data management {19}

Three forms are collected and checked at each follow-up visit and then stored securely in the trial locker: two patient-reported outcomes measure (PROM), the OMAS and RAND-36, and the case report form. All data are transferred to a temporal database located within the hospital computer system by two researchers every 2 weeks, and a read-only copy is stored in a separate folder. This is then cross-checked before data are transferred to the statistical software for statistical analysis and reporting by the statistician and the research team.

The RAND-36 requires multiple steps analysis; this will be performed with oblique scoring and the orthogonal factor analytic model [24]. Normative data for the Irish population will be used as a reference [25].

#### Confidentiality {27}

Data management will be in accordance with the *General Data Protection Regulation* (GDPR) Health Service Executive (HSE) and Health Research regulations [26]. Data will be kept anonymously in the HSE local hospital computer system database in protected folders to ensure confidentiality. Paperwork will be stored in the trial locker in a locked researcher office within the hospitals.

#### Plans for collection, laboratory evaluation and storage of biological specimens for genetic or molecular analysis in this trial/future use {33}

Not applicable as there is no laboratory evaluation or biological specimen collection in this study.

### Statistical methods

#### Statistical methods for primary and secondary outcomes {20a}

Continuous outcomes and other key variables will be described by their means and SDs, medians and IQRs, and total range. Categorical variables will be described by counts and respective proportions.

The primary outcome, OMAS score at 6 weeks, will be analysed using multiple linear regression with fixed effects for study arm and centre, from which we will report the centre-adjusted difference in mean OMAS scores between the two arms with a 95% confidence interval (CI) and exact p value. The complete set of longitudinal OMAS scores, from 2 weeks to 1 year, will be analysed with the corresponding linear mixed-effects model (with the identity link function), with fixed effects for the centre, arm, and time (dummy coded) and an interaction term between arm and time. Time-specific differences in the mean OMAS scores between arms will be calculated from the model’s results, along with 95% CIs and the exact p value from the time by arm interaction.

Longitudinally measured secondary outcomes will be similarly analysed and reported: ankle range of motion with the same linear mixed-effects model and RAND-36 scores with an ordinal mixed-effects model with a logit link function (i.e. the proportional odds model). Time to return to work (in the subset working at baseline) and length of stay will be analysed using Cox proportional hazards models, with fixed effects for centre and treatment arm. Infection (any) and fixation failure at any point during study follow-up will be similarly analysed using logistic regression.

There will be no formal adjustments for multiplicity, but we will report all estimated treatment effects alongside exact p values, allowing the reader to make whatever adjustments they prefer. Any deviations from the above plan will be fully described and justified in the final report of the trial results. All analyses and the production of tables and plots will be conducted using the R (version 3.6.3, R Project for Statistical Computing) and Stata16 software. All analyses will be conducted or supervised by the principal statistician of the HRB Clinical Research Facility Cork under their quality system and relevant standard operating procedures and following regulatory guidance (e.g. ICH E9).

Because outcomes at week 6 are collected as part of routine care, we do not anticipate and intentionally aim to avoid any missing values at that time point. However, if there are missing values, we will carefully consider why data are missing and employ appropriate methods, which could range from complete case analysis in the presence of very little missing data deemed MCAR to multiple imputations under assumptions of MAR. We will take advantage of full information maximum likelihood estimation for the mixed-effects models to retain all patients in the models for missing longitudinal data. Irrespective of the actual approach taken (which cannot be optimally decided on without consideration of the actual study data), we will explore the choices and their potential impact on inferences with sensitivity analyses.

#### Methods for additional analyses (e.g. subgroup analyses) {20b}

No planned additional analysis.

#### Methods in analysis to handle protocol non-adherence and any statistical methods to handle missing data {20c}

Any protocol non-adherence will be disclosed and handled accordingly. An effort will be made to prevent missing data as much as possible. Unavoidable missing data, such as withdrawals from the study or loss to follow-up data, will be analysed on an intention-to-treat basis, including sensitivity analysis. Multiple imputations using chain equations (MICE) will be used for missing data [27–29].

#### Plans to give access to the full protocol, participant level-data and statistical code {31c}

The full protocol is available at the registry website and will be published in one of the trial protocol journals.

### Oversight and monitoring

#### Composition of the coordinating centre and trial steering committee {5d}

Four authors at the coordinating centre (University Hospital Waterford) take responsibility for the scientific validity of the study protocol, assessment of study quality and conduct, as well as for the scientific quality of the final study report.

#### Composition of the data monitoring committee, its role and reporting structure {21a}

The authors understand the composition for a standard data monitoring committee (DMC) for this trial is challenging and might be impossible. The lack of funding is the main barrier. Unlike a clinical trial where a DMC and interim analysis must be formulated, this non-interventional trial, with relatively small numbers, does not investigate a pharmacological or medicinal product and does not expose patients to significant harm. Both treatment methods are already part of routine practice. As such, the trial team will closely monitor any adverse event or harm that might arise and act accordingly on a daily basis (see below).

#### Interim analyses {21b}

No planned interim analysis.

#### Adverse event reporting and harms {22}

Collected case report forms will be checked daily by the research team before being stored in the trial locker; any adverse events or harm, such as failure of fixation, DVT or surgical site complications, will be communicated with the study team.

#### Frequency and plans for auditing trial conduct {23}

The trial conduct is continuously audited in the departmental audit meeting (3 monthly).

#### Plans for communicating important protocol amendments to relevant parties (e.g. trial participants, ethical committees) {25}

Any change to the trial protocol will be communicated with the ethical committees and trial registry.

#### Dissemination plans {31a}

The results of this trial will be published in one of the peer-reviewed medical journals.

## Discussion

The National Institute for Health and Care Excellence (NICE) has recommended the subject as worthy of further research [30], and this has been reinforced by a recent audit of the UK Practice [3]. Furthermore, the latest systematic review and meta-analysis by Smeeing et al. in 2015 reviewed the effect of early mobilisation and early weight-bearing. Analysis of short- and long-term functional outcomes after weight-bearing was not possible due to the lack of studies and proper reporting. Furthermore, only three studies with a total number of 67 patients were included in the return to work analysis and had substantial heterogeneity [21].

We performed a systematic literature review and meta-analysis of RCTs and comparative cohort studies, which will be published soon. The protocol is registered to PROSPERO [31]. To our knowledge, immediate weight-bearing (IWB) following ORIF of all types of unstable ankle fractures has not been investigated in a controlled prospective manner in recent decades. This will be the largest pragmatic randomised-controlled multicentre trial that examines the safety and efficacy of IWB following ORIF of all ankle fracture patterns in the usual condition of care and will help in formulating a widely accepted guideline for postoperative management of ankle fractures.

## Trial status

At the time of the initial manuscript submission, 90 patients were recruited. Recruitment started on 7 January 2019 and completed on 11 June 2020. A 1-year follow-up is planned. This protocol is the 6th version and dated 20 June 2020.

## Supplementary Information

**Additional file 1.** Patient information leaflet.

**Additional file 2.** Postoperative care information sheet (cast).

**Additional file 3.** Postoperative care information sheet (boot).

**Additional file 4.** Case Report Form.

## Data Availability

No personal data will be available. The coded datasets used and analysed during this trial will be available from the corresponding author on reasonable request following the GDPR and Health Research Regulations 2018 [26].

## Abbreviations

ORIF: Open reduction and internal fixation
IWB: Immediate weight-bearing
ROM: Range of motion
NWB: Non-weight-bearing
OMAS: Olerud-Molander Ankle Score
EWB: Early weight-bearing
HSE: Health Service Executive
LOCF: Observation carried forward
BOCF: Baseline observation carried forward
GDPR: General Data Protection Regulation
DMC: Data Monitoring Committee
ITT: Intention-to-treat
