# Correction to: Is postoperative non-weight-bearing necessary? INWN Study protocol for a pragmatic randomised multicentre trial of operatively treated ankle fracture

**DOI:** 10.1186/s13063-021-05368-5

**Published:** 2021-06-21

**Authors:** Ramy Khojaly, Ruairí Mac Niocaill, Muhammad Shahab, Matthew Nagle, Colm Taylor, Fiachra E. Rowan, May Cleary

**Affiliations:** 1 Department of Trauma and Orthopaedic Surgery, University Hospital Waterford, Waterford, X91 ER8E Ireland; 2 Department of Surgery, Royal College of Surgeons in Ireland, Dublin, D02 YN77 Ireland; 3 Department of Orthopaedic Surgery, University College Cork, Cork, T12 YN60 Ireland; 4 Department of Trauma and Orthopaedic Surgery, Cork University Hospital, Cork, T12 DFK4 Ireland

**Correction to: Trials 22, 369 (2021)**

**https://doi.org/10.1186/s13063-021-05319-0**

Following the publication of the original article [1], we were notified that in the Administrative Information table (Author details 5a), the numbering and the order of the institution was incorrect.

The original article has been corrected.
