# Supplementary material for: Is postoperative non-weight-bearing necessary? INWN Study protocol for a pragmatic randomised multicentre trial of operatively treated ankle fracture
Source: Trials. 2021 May 27;22:369. doi: 10.1186/s13063-021-05319-0 (PMC8161990; doi:10.1186/s13063-021-05319-0)
Supplement: Supplementary file 1 — Additional file 1. Patient information leaflet. [file 13063_2021_5319_MOESM1_ESM.pdf]

Ankle Study Patient Information Leaflet

Study title: Do we have to keep patients in a cast and prevent them weight-bearing following surgical repair of ankle fractures?

Trial Number: ISRCTN76410775

Lead co-investigators: Prof. Ruairi MacNiocaill, Prof. May Cleary, Prof. Stephen Kearns, Mr Colm Taylor

Principal investigator: Mr. Ramy Khojaly
Telephone number of the principal investigator: (051) 842 198
E-mail of principal investigator: ramykhojaly@rcsi.com

Data Controller's/joint Controller's Identity: Mr.Ramy Khojaly
Data Controller's/joint Controller's Contact Details: ramykhojaly@rcsi.com

You are being invited to take part in a research that is carried out by the Orthopaedic team at University Hospital Waterford, Cork University Hospital and Galway University Hospital.
Before you decide whether or not you wish to take part, you are advised to read the information provided below carefully. If you want, discuss it with your family, friends or GP (doctor). Take time to ask questions – don't feel rushed and don't feel under pressure to make a quick decision. You should clearly understand the risks and benefits of taking part in this study so that you can make a decision that is right for you. This process is known as 'Informed Consent'.
You don't have to take part in this study. You can change your mind about taking part in the study at any time you like. Even if the survey has started, you can still opt-out. You don't have to give us a reason. If you do opt-out, rest assured it won't affect the quality of treatment you get in the future.

Why is this study being done?

This study is aiming to compare the outcome of two groups of patients after fixation of an ankle fracture. The first group will get a cast after surgery, and they will not be allowed to weight bear for six weeks. The second group will get a walking boot and will be allowed to weight bear as tolerated.

Who is organising and funding this study?

A group of orthopaedic surgeons conducts this research. The funding for this research is self-funded by the principal investigator and no payment granted to recruit patients for the study.

Why am I being asked to take part?

You are being asked to take part because you have an ankle fracture that will need surgical fixation.

How will the study be carried out?

This study commenced in January 2019; more than 100 patients will be recruited to take part in this study.

What will happen to me if I agree to take part?

As part of this study, you can expect either to have a below-knee cast (the standard practice) or a walking boot (the study subject) after surgery. If a cast is applied, you will not be allowed to weight bear on your fractured ankle for six weeks, and you will walk using crutches. But, if a walking boot is applied, you will start weight-bearing as you tolerate with the help of crutches and progress to full weight-bearing as soon as you can. You will also be allowed to remove the boot after two weeks to do some ankle exercise.

It is essential to elevate the foot in the first two weeks to allow the swelling to reduce and the surgical wound to heal.

After two weeks, the first outpatient appointment will be for removal of sutures or clips and to have an x-ray. The second appointment will be in 6 weeks to have another x-ray after removal of the cast or boot.

What we know from previous studies, both ways of treatments are safe. The application of walking boot and allowing weight bearing doesn’t increase the risk of complications. Although, more studies are needed in this field to prove if the outcome will be better in changing the common practise, that why you have been asked to be part of this study.

To examine your progress, the research team have to review your medical records.

What are the benefits?

There is only a few similar research undertaken in this subject. Early weight-bearing makes no differences in terms of wound complications, surgical site infections, or loss of fracture fixation. Improved early functional outcome in term of ankle movement is reported.

What are the risks?

The risk and complications of surgical fixations of ankle fractures apply to both groups of patients. The common complications are; wound complications including infection, deep infection, numbness around the surgical site, failure of the body to heal the fracture (non-union), which may lead to failure of fixation, ankle joint arthritis as a result of ankle fracture and deep vein thrombosis.

Prophylactic antibiotics to reduce the risk of infection will be given to you before and after surgery. Also, blood thinning medication could be prescribed as indicated.

Is the study confidential?

As part of this research, the research team will be looking at all the participated patient's records to monitor their treatment and to compare outcomes. Your information will be kept private and confidential all the time. At the end of the study, the final result will be published in medical journals and presented in medical conferences with no personal information.
If you would like to know about the result of this study, please contact the principal investigator.

Where can I get further information?

If you need any further information now or at any time in the future, please contact
Mr Ramy Khojaly E-mail Address: ramykhojaly@rcsi.com
Phone No: (051) 842 198

Data Protection

1. The purpose or reason for processing your data in our research to help us study if changing the treatment protocol will improve patients outcome.
2. The legal basis under which we are processing your data is for scientific research purposes
3. The research team will have access to the research participants' information.
4. The data will be stored for approximately one year or until the research end.
5. No risks and implications are expected as a result of data processing.
6. You have the right to withdraw consent to your data being used in this research project. You will be able to do this by contacting Mr Ramy Khojaly by sending an e-mail to ramykhojaly@rcsi.com.
7. You have a right to complain with the Data Protection Commissioner.
8. You have a right to request access to your data and a copy of it unless your request would make it impossible or make it very difficult to conduct the research.
9. You have a right to restrict or object to processing unless your request would make it impossible or make it very difficult to conduct the research.
10. You have a right to have any inaccurate information about you corrected or deleted unless your request would make it impossible or make it very difficult to conduct the research.
11. You have a right to have your data deleted unless your request would make it impossible or make it very difficult to do the research.
12. You have a right to move your data from one controller to another in a readable format.
13. There will be no automated processing of personal data to evaluate certain personal aspects relating to you.
14. You have a right to object to automated processing, including profiling if they wish.
15. Your data will not be further processed for other purposes.
16. Your data will not be transferred to another country.
