# Supplementary material for: Is postoperative non-weight-bearing necessary? INWN Study protocol for a pragmatic randomised multicentre trial of operatively treated ankle fracture
Source: Trials. 2021 May 27;22:369. doi: 10.1186/s13063-021-05319-0 (PMC8161990; doi:10.1186/s13063-021-05319-0)
Supplement: Supplementary file 2 — Additional file 2. Postoperative care information sheet (cast). [file 13063_2021_5319_MOESM2_ESM.pdf]

UHW
Ospidéal Ollscoile Phort Láirge
University Hospital Waterford

UCC
Coláiste na hOllscoile Corcaigh, Éire
University College Cork, Ireland

CUH
Ospidéal na hOllscoile Corcaigh
Cork University Hospital

Galway University Hospitals
Ospidéil na h-Ollscoile Gaillimh
UNIVERSITY HOSPITAL GALWAY
MERLIN PARK UNIVERSITY HOSPITAL

RCSI

# Ankle Trial: Post-operative instruction

## Cast Group

**Study Title:** Do we have to keep patient in a cast and prevent them from weight-bearing following internal fixation of ankle fractures?

**Trial Number**: ISRCTN76410775 **Sites Lead-investigators:** Prof. Ruairi MacNiocaill, Prof. May Cleary, Mr Colm Taylor, Prof. Stephen Kearns

***Principal investigator:*** *Mr* Ramy Khojaly

1. Please keep your foot elevated most of the time in the first two weeks to reduce swelling and to enhance wound healing.
2. Avoid wetting your cast as this might lead to skin irritation and wound complication.
3. Please use crutches or frame to walk, and avoid putting any weight through your fractured ankle until the fracture heals in about six weeks.
4. Your first outpatient follow up will be in about two weeks; an appointment will be arranged and sent to your address.
5. At your first appointment, you will have a check x-ray, your wound will be checked, your clips or sutures will be removed. Another cast will be applied, and that will stay for a further four weeks.
6. Your second appointment will be six weeks after your surgery, and the cast will be removed then, you will have a check x-ray. At this stage, you will start weight-bearing and physiotherapy.
7. Please be assured that it is normal for your ankle to be stiff after being in the cast for six weeks. The doctor will give you a physiotherapy request form at this visit.
8. Follow up appointments will be arranged as following; 3 months, six months and one year following surgery, you will have a check X-ray at each visit.
9. At each visit, please complete the two questioner forms that will be provided by the opd nurses and give it back to the nurses/doctors.
