# Supplementary material for: Is postoperative non-weight-bearing necessary? INWN Study protocol for a pragmatic randomised multicentre trial of operatively treated ankle fracture
Source: Trials. 2021 May 27;22:369. doi: 10.1186/s13063-021-05319-0 (PMC8161990; doi:10.1186/s13063-021-05319-0)
Supplement: Supplementary file 3 — Additional file 3. Postoperative care information sheet (boot). [file 13063_2021_5319_MOESM3_ESM.pdf]

Ankle Trial: Post-operative instruction

Walking Boot Group

Study Title: Do we have to keep patient in a cast and prevent them weight-bearing following internal fixation of ankle fractures?

Trial Number: ISRCTN76410775 **Sites Lead-investigators:** Prof. Ruairi MacNiocaill, Prof. May Cleary, Mr Colm Taylor, Prof. Stephen Kearns

Principal investigator: *Mr* Ramy Khojaly

1. Please keep your foot elevated most of the time in the first two weeks to reduce swelling and to enhance wound healing.
2. You are allowed to walk while wearing the walking boot. You have to use crutches or walking frame, you can weight bear on your foot as much as you tolerate.
3. Your physiotherapy starts from the first day after surgery, please remove the walking boot five times a day for gentle ankle exercises, keep the boot on at night. Please do not remove the inner dressing at the surgical site and keep it dry all the time.
4. Your first outpatient follow up will be in about two weeks; an appointment will be arranged and sent to your address.
5. At your first appointment, you will have a check x-ray, your wound will be checked, your clips or sutures will be removed. The walking boot will be re-applied, which will stay for a further four weeks. The doctor will give you a physiotherapy referral form at this visit, and you can continue to weight bear as you tolerate.
6. Your second appointment will be six weeks after the surgery. The walking boot will be removed and then you will have a check x-ray to confirm fracture healing, at this stage you can wear your normal shoes.
7. Follow up appointment will be arranged as following; 3 months, six months and one year following surgery, you will have a check X-ray at each visit.
8. At each visit, please complete the two questioner forms that will be provided by the opd nurses and give it back to the nurses/doctors.

UHW

Ospidéal Ollscoile
Phort Láirge
University Hospital
Waterford

UCC
Coláiste na hOllscoile Corcaigh, Éire
University College Cork, Ireland

CUH
Ospidéal na hOllscoile Corcaigh
Cork University Hospital

Galway
University
Hospitals
Ospidéil na h-Ollscoile Gaillimh
UNIVERSITY HOSPITAL GALWAY
MERLIN PARK UNIVERSITY HOSPITAL

RCSI
