# Supplementary material for: Is postoperative non-weight-bearing necessary? INWN Study protocol for a pragmatic randomised multicentre trial of operatively treated ankle fracture
Source: Trials. 2021 May 27;22:369. doi: 10.1186/s13063-021-05319-0 (PMC8161990; doi:10.1186/s13063-021-05319-0)

Patient Sticker

UHW | Ospidéal Ollscoile Phort Láirge | University Hospital Waterford | UCC | CUH Cork University Hospital | Galway University Hospitals | RCSI

Ankle Trial Case Report Form

Dear Doctor, please tick the appropriate box and write your comment if any at each follow up visit. Please keep the form in the provided box in the clinic.

Date: Patient Name: MRN:

Time since surgery	2 weeks ☐<br>6 weeks ☐<br>12 weeks ☐<br>6 months ☐<br>1 year ☐	H/O Diabetes	Yes ☐<br>No ☐
		Surgeon	Consultant ☐<br>Registrar ☐
Surgical site examination<br>Comment and other complications:	Healed ☐<br>Infected ☐<br>Dehiscence ☐<br>Other ☐	X-ray<br>Comment:	Ok ☐<br>Union ☐<br>Non-union ☐<br>Fixation failure ☐
Ankle Dorsiflexion<br><br>Please write the exact Goniometer measure……..	-5 ☐<br>0 ☐<br>5 ☐<br>10 ☐<br>20 ☐	Ankle Plantarflexion<br><br>Please write the exact Goniometer measure…….	10 ☐<br>20 ☐<br>30 ☐<br>40 ☐<br>45 ☐
Return to work<br>Date:…/…/20…..	Yes ☐<br>No ☐	Have you referred the patient to Physio?	Yes ☐<br>No ☐
Olerud-Molander score completed and collected?	Yes ☐<br>No ☐	SF-36 Health Survey completed and collected?	Yes ☐<br>No ☐

<u>Guidance for Ankle ROM Goniometer measure</u>

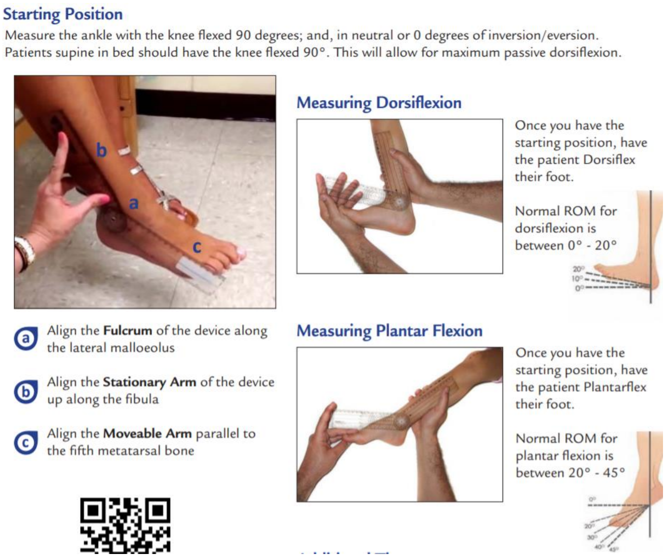

Supplement: Supplementary file 4 — Additional file 4. Case Report Form. [file 13063_2021_5319_MOESM4_ESM.pdf]
